# The American Institute of Homœopathy: Semi-Centennial 1844–1894

**Published:** 1894-07

**Authors:** 


THE AMERICAN INSTITUTE OF HOMŒOPATHY: Semi-Centennial 1844–1894. Section of Materia Medica and General Therapeutics; Programme of the Section. Frank Kraft, M. D., Cleveland, Ohio, Chairman; Wm. E. Leonard, M. D., Minneapolis, Secretary.

This little book of one hundred and ten pages is an admirable little compend of the special work of the section.

It contains the order of exercises and a series of questions asked of the members of the section by the Chairman, with the answers thereto.

It is embellished by photo-gravure portraits of the members of the section, and is altogether a tasteful and handy little volume. Of course, it is the work of Dr. Frank Kraft, the Chairman of the section, well known to the profession in general as the clever editor of *The American Homœopathist.*

Dr. Kraft is distinguishing himself by his efforts to improve the study of materia medica, and to make the study more universal.

In the pride of intellect that dominates the majority of the members of our school, they have given their attention to rational therapeutics to the exclusion of the materia medica, which they have treated with contempt. The result is the students in the colleges have gotten but small instruction in the branch that is the corner-stone of the school. Dr. Kraft, by his original way of attacking the problem, has revived the study of materia medica and improved the methods.

